# Predictive Analysis of Drug-Resistant Tuberculosis: Integrating Molecular Markers, Clinical Governance, and Community-Engaged Education in Rural South Africa

**DOI:** 10.3390/diseases14040132

**Published:** 2026-04-03

**Authors:** Siphosihle Conham, Ncomeka Sineke, Ntandazo Dlatu, Lindiwe Modest Faye, Mojisola Clara Hosu, Teke Apalata

**Affiliations:** 1 WSU—TB Research Group, School of Pathology, Faculty of Medicine and Health Sciences, Walter Sisulu University, Private Bag X1, Mthatha 5100, South Africa; 221226184@mywsu.ac.za (S.C.); 209101237@mywsu.ac.za (N.S.); mhosu@wsu.ac.za (M.C.H.); tapalata@wsu.ac.za (T.A.); 2 Walter Sisulu Institute for Clinical Governance, Healthcare Administration, School of Public Health, Faculty of Medicine and Health Sciences, Walter Sisulu University, Mthatha 5099, South Africa; ndlatu@wsu.ac.za

**Keywords:** drug-resistant tuberculosis, machine learning, predictive modelling, *katG*, *rpoB*, clinical governance, community-engaged education

## Abstract

Background: Drug-resistant tuberculosis remains a major challenge in resource-limited settings, particularly in rural regions of the Eastern Cape Province, where limited laboratory infrastructure, constrained access to advanced molecular diagnostics, shortages of specialized healthcare personnel, and prolonged diagnostic turnaround times can delay appropriate treatment initiation. This study examined whether routinely detectable genomic resistance markers could be integrated with parsimonious machine learning approaches to support early risk stratification for isoniazid (INH) and/or rifampicin (RIF) resistance and multidrug-resistant tuberculosis (MDR-TB). Methods: We conducted a retrospective analysis of clinical, demographic, and genomic data from 207 *Mycobacterium tuberculosis* isolates representing 207 unique patients. Resistance was classified as INH and/or RIF resistance or MDR-TB (concurrent resistance to both drugs). Predictors included age, sex, and canonical resistance-associated mutations (*katG* S315T, *inhA* −15C>T, and *rpoB* codon substitutions). Logistic regression was used to estimate adjusted odds ratios (aORs), while Random Forest models were applied to assess non-linear feature importance. Internal validation was performed using 10-fold cross-validation. A systems network analysis mapped the integration of model-derived risk bands into Clinical Governance structures and Community-Engaged Education pathways, including interventions delivered by Community Health Workers (CHWs). Results: INH and/or RIF resistance was identified in 58.9% of isolates, with 21.7% classified as MDR-TB. The most frequently detected mutations were *katG* S315T (29.0%) and *rpoB* S450L (26.6%). Logistic regression identified *rpoB* S450L (aOR 4.20; 95% CI: 2.10–8.45) and *katG* S315T (aOR 2.85; 95% CI: 1.40–5.80) as the strongest independent predictors, while age and sex were not statistically significant. Models demonstrated strong internal discrimination (AUCs of 0.96 for INH and/or RIF resistance and 0.99 for MDR-TB). Risk stratification categorized 18% of patients as high risk. Scenario-based modelling suggested that prioritizing high-risk patients for reflex Line Probe Assay testing could reduce the median time to appropriate treatment from 14 to 3 days and may reduce progression from isoniazid-resistant TB to MDR-TB under specified operational assumptions. Conclusions: Mutation-informed predictive modelling demonstrates strong internally validated discrimination and provides a structured framework for risk-stratified intervention. Integrating probability-based risk thresholds within Clinical Governance systems and community-level support structures, including CHW-led adherence and education strategies, may support earlier treatment optimization in high-burden rural settings. External validation and prospective implementation studies are required before broader programmatic adoption.

## 1. Introduction

Tuberculosis (TB) remains one of the most significant infectious diseases worldwide, with drug-resistant tuberculosis (DR-TB) representing a major threat to public health, especially in high-burden and resource-limited settings [1,2]. According to the latest WHO Global Tuberculosis Report, millions of people develop TB each year, and multidrug-resistant TB (MDR-TB), defined as resistance to both isoniazid and rifampicin, accounts for a large share of treatment failures and TB-related deaths globally [3]. South Africa continues to be a key contributor to the global burden of DR-TB. In this context, ongoing transmission of resistant strains, combined with delays in starting appropriate treatment, sustains transmission, particularly in rural provinces with limited healthcare infrastructure, workforce shortages, and barriers to prompt diagnosis [4,5,6]. The biological foundation of drug resistance in *Mycobacterium tuberculosis* is well understood and primarily driven by chromosomal mutations that modify drug targets or affect enzyme activation pathways. Resistance to isoniazid is most often linked to mutations in the *katG* gene, especially the S315T substitution, which confers high-level resistance, as well as promoter mutations in *inhA* (e.g., −15C>T), which are associated with low-level resistance [7,8,9,10,11]. Rifampicin resistance mainly results from mutations within the rifampicin resistance-determining region (RRDR) of the *rpoB* gene, with the S450L substitution among the most reported high-confidence mutations worldwide [7,8,12]. The WHO Catalogue of Mutations in *Mycobacterium tuberculosis* Complex and Their Association with Drug Resistance (2nd edition) details a broad spectrum of resistance-related variants across these loci. However, routine molecular diagnostics employed in many high-burden settings, including GeneXpert and Line Probe Assays (LPAs), detect only a subset of these high-confidence mutations [13,14,15]. In resource-limited environments, clinical and programmatic decisions are often based on these routinely detectable canonical mutations rather than on comprehensive whole-genome sequencing data. Understanding how these commonly detected resistance markers can aid early risk assessment and targeted interventions remains operationally critical. Delays in detecting resistance lead to inappropriate initial therapy, prolonged infectiousness, treatment escalation, and the progression from isoniazid-resistant TB to MDR-TB [16,17]. Isoniazid-resistant TB is especially troubling because it often precedes additional resistance, creating a vital window for intervention before full multidrug resistance develops. Recent advances in predictive analytics and machine learning (ML) have shown promise for enhancing resistance prediction and risk stratification in TB [18,19,20,21,22]. However, when predictor sets are biologically deterministic and relatively simple, centred around canonical resistance mutations, the added benefit of complex algorithms over interpretable statistical models may be limited [23]. Moreover, few studies have translated predictive modelling into structured operational frameworks such as Clinical Governance (CG) systems or Community-Engaged Education (CEE) initiatives, especially in rural African health systems where governance gaps, stigma, and delayed care-seeking hinder TB control efforts [24,25,26]. This study addresses these gaps by developing and internally validating a mutation-based predictive framework focused on high-confidence resistance markers (*katG*, *inhA*, and *rpoB* variants) that are routinely detectable in the study setting. Instead of modeling the entire mutation spectrum described in the WHO catalogue, the analysis focuses on variants detectable by standard diagnostic methods to ensure operational relevance and statistical robustness. The predictive outputs are then mapped onto structured CG pathways and risk-based CEE interventions to demonstrate how molecular evidence could facilitate timely regimen adjustments, reflex testing strategies, and household-level prevention. By aligning mutation-based predictions with governance accountability and community engagement mechanisms, this study offers a structured, context-specific approach to enhance DR-TB control in high-burden rural environments.

## 2. Materials and Methods

### 2.1. Study Design and Setting

A quantitative, analytical cross-sectional study was conducted using routinely collected TB program data from public health facilities in the Eastern Cape Province, South Africa. Facilities were selected based on high notification rates of isoniazid- and/or rifampicin-resistant tuberculosis and the availability of molecular diagnostic records.

This study utilised secondary programmatic data collected under routine clinical care, and no additional diagnostic procedures or interventions were introduced. The use of real-world program data enhances the external relevance of the findings to similar high-burden, resource-limited settings.

### 2.2. Study Population, Sampling, and Sample Size Justification

A census sampling strategy was employed, whereby all eligible cases meeting the inclusion criteria during the study period (January 2018–March 2020) were included in the analysis. The final analytical sample comprised 207 patients with laboratory-confirmed pulmonary TB No formal a priori sample size calculation was performed due to the retrospective census design. However, sample adequacy for predictive modeling was assessed using the events-per-variable (EPV) principle. The dataset included 45 MDR-TB events, and the final model incorporated a limited number of predictors, ensuring an EPV ratio consistent with recommended thresholds (>10 events per predictor variable). This approach reduces the risk of overfitting and supports model stability.

### 2.3. Data Collection and Data Quality Control

Data were obtained from multiple routine health information systems, including EDRWeb, laboratory diagnostic platforms, and the District Health Information System (DHIS). To ensure data quality and integrity, a systematic verification process was undertaken. Data from different sources were cross validated to confirm consistency, and all records were carefully reviewed for completeness and internal coherence. Implausible or inconsistent values were identified through logical checks and were corrected where appropriate based on source records. All data were fully anonymised prior to analysis, and no identifiable patient information was accessible at any stage of the study, thereby ensuring confidentiality and compliance with ethical standards.

### 2.4. Predictor Variables

Predictor variables were selected based on their biological relevance, availability within routine programmatic data, and overall completeness within the dataset. Priority was given to well-established resistance-associated mutations with strong evidence linking them to drug resistance mechanisms. In addition, variables routinely captured in program settings were favoured to enhance the model’s practical applicability and reproducibility in similar resource-limited contexts. Consideration of data completeness was also critical in the selection process to minimise bias and ensure robustness of the modelling framework. As a result, only variables with sufficiently reliable and consistent data were retained for inclusion in the final predictive models. The analysis was restricted to high-confidence mutations that are routinely detectable in programmatic diagnostic settings. Rare mutations were not modelled separately due to their low frequency and the potential for unstable estimates in a modest sample size. This approach ensured model interpretability, robustness, and applicability in real-world clinical settings.

#### Handling of Missing Data

Several clinically relevant variables (e.g., BMI, diabetes, treatment delay) showed substantial missingness or inconsistent documentation. Given the modest sample size and the risk of bias associated with extensive imputation, these variables were excluded from the predictive modeling. A complete-case analysis approach was adopted for variables included in the final model.

### 2.5. Outcome Variables

Outcome classification followed standardized World Health Organization (WHO) definitions, ensuring consistency with international reporting frameworks. Treatment outcomes were categorised as successful or unsuccessful according to established criteria. For analytical purposes, unsuccessful outcomes were aggregated into a binary variable. This approach was adopted to maintain adequate statistical power and to minimise instability arising from small sample sizes within individual outcome subgroups. The binary classification further facilitated robust logistic modelling while preserving alignment with programme reporting standards.

### 2.6. Data Analysis and Model Development

#### 2.6.1. Model Specification and Assumptions

Binary logistic regression was specified a priori as the primary modeling approach due to its interpretability and suitability for low-dimensional clinical datasets. Key model assumptions were systematically assessed to ensure validity of the analysis. Multicollinearity among predictors was evaluated using variance inflation factors (VIFs), while the assumption of linearity in the logit was examined for continuous variables, particularly age. Independence of observations was ensured by restricting the dataset to unique patient records, thereby preventing duplication and clustering effects.

#### 2.6.2. Overfitting Control

To minimise the risk of overfitting, model complexity was deliberately restricted through the use of a parsimonious set of predictors. Variable selection was guided by biological plausibility and data completeness to ensure both interpretability and robustness. In addition, cross-validation techniques were employed to evaluate model stability and reduce optimism bias in performance estimates.

#### 2.6.3. Internal Validation

Internal validation was conducted using stratified 10-fold cross-validation, which preserved the proportional distribution of outcome classes across training and validation datasets. Preprocessing steps were applied within each training fold and subsequently extended to the corresponding validation fold, thereby preventing data leakage. Performance metrics were averaged across all folds to generate stable and internally validated estimates of model performance.

#### 2.6.4. Model Performance and Uncertainty

Model performance was evaluated using both discrimination and calibration metrics. Discrimination was assessed through measures such as the area under the receiver operating characteristic curve, while calibration was examined by comparing predicted probabilities with observed outcomes.

However, it is important to note that model outputs and scenario-based projections represent deterministic estimates and do not incorporate uncertainty intervals or probabilistic sensitivity analyses. As such, the findings should be interpreted cautiously as proof-of-concept results rather than definitive predictive tools.

### 2.7. Reporting Standards

This study was reported in accordance with the TRIPOD (Transparent Reporting of a multivariable prediction model for Individual Prognosis or Diagnosis) guidelines, ensuring transparency in model development, validation, and reporting.

### 2.8. Conceptual Integration with Clinical Governance and Community Engagement

To illustrate potential operational pathways, model-derived risk categories were mapped onto existing facility-level CG structures and CEE strategies. Within CG systems, risk classification was conceptually linked to predefined Standard Operating Procedures (SOPs) for reflex molecular testing, risk-stratified regimen review, and enhanced contact tracing. Key performance indicators (KPIs), including time to appropriate regimen initiation and household screening coverage, were identified as metrics for incorporation into routine audit-and-feedback cycles. At the community level, the framework aligned risk stratification with differentiated CEE interventions delivered through community health workers (CHWs). High-risk profiles were associated with intensified household engagement, adherence monitoring, and contact tracing, whereas moderate-risk profiles were associated with enhanced counselling and follow-up support. Low-risk profiles were linked to routine TB education and standard community outreach. This integration framework is intended to demonstrate how predictive outputs could theoretically be embedded within existing governance and community systems. It represents a conceptual implementation illustration rather than an evaluated operational model, and further implementation research is required to assess feasibility, acceptability, and effectiveness in routine practice.

### 2.9. Scenario Simulations

To explore potential programmatic implications of risk-guided intervention, deterministic scenario simulations were conducted comparing two conceptual pathways:

1. Standard care pathway, reflecting observed programmatic diagnostic delays (median delay ≈14 days);
2. Predictive-reflex pathway, in which high-risk classifications trigger rapid molecular testing and earlier regimen review.

Two projected outcomes were examined:

1. Potential reduction in the median time to initiation of an appropriate drug regimen;
2. The proportion of isoniazid-resistant TB cases potentially prevented from progressing to MDR-TB through earlier detection and regimen optimization.

Simulations were based on observed baseline diagnostic delays and predefined operational assumptions, including rapid reflex LPA turnaround (≤72 h) and prompt regimen adjustment following detection of resistance-associated mutations. These analyses represent deterministic projections under simplified operational assumptions and did not incorporate stochastic transmission modelling, probabilistic sensitivity analyses, or uncertainty intervals. Consequently, projected reductions in diagnostic delay and resistance progression should be interpreted as theoretical estimates intended to illustrate potential programmatic impact rather than empirical intervention outcomes.

## 3. Results

### 3.1. Phenotype Distribution

Among 207 isolates (Table 1), 122 (58.9%) exhibited resistance to isoniazid and/or rifampicin. MDR-TB (resistance to both isoniazid and rifampicin) accounted for 45 cases (21.7%). Rifampicin monoresistance was detected in 28 isolates (13.5%), and isoniazid monoresistance in 36 isolates (17.4%). Eighty-five isolates (41.1%) were fully susceptible to both isoniazid and rifampicin.

### 3.2. Prevalence of Key Resistance Mutations

The most frequently detected resistance-associated mutation was *katG* S315T (29.0%), followed by *rpoB* S450L (26.6%) and the *inhA* promoter mutation −15C>T (20.3%). Additional *rpoB* codon substitutions were observed at H445 (8.7%) and D435 (5.8%) (Table 2).

Dual INH-associated mutations (*katG* S315T + *inhA* −15C>T) were identified in 28 isolates (13.5%), predominantly among isolates classified as isoniazid-resistant TB or MDR-TB.

Among rifampicin resistance-associated mutations evaluated in this dataset, *rpoB* S450L was the most frequently detected variant, occurring in 40 isolates (19.3%) classified as rifampicin-resistant or MDR-TB (Table 3).

Isolates with isolated *katG* S315T or *inhA* −15C>T mutations were primarily associated with isoniazid monoresistance (10.6% and 6.8%, respectively). All isolates without detected mutations were phenotypically susceptible to both drugs (41.1%).

### 3.3. Diagnostic Machine Learning Performance

**Figure 1:**
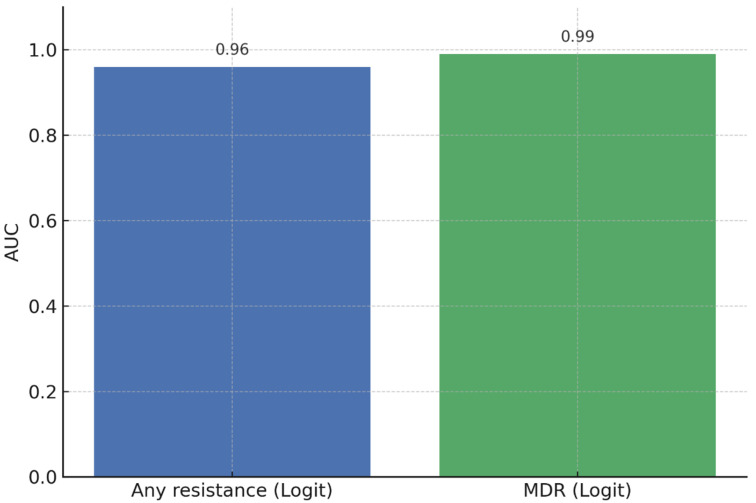
Diagnostic ML performance (logistic regression).

Logistic regression and Random Forest classifiers were used with age, sex, and mutation indicators (*katG*, *inhA*, and *rpoB* variants) as predictors. Logistic regression showed strong internal discrimination, with an AUC of 0.96 for isoniazid and/or rifampicin resistance and 0.99 for MDR-TB (Figure 1). Calibration plots indicated close agreement between predicted probabilities and observed outcome frequencies across risk deciles. The mean Brier score across 10-fold cross-validation reflected acceptable probabilistic accuracy.

Receiver operating characteristic (ROC) curves showing the diagnostic performance of the logistic regression model for predicting isoniazid and/or rifampicin resistance and MDR-TB.

**Figure 2:**
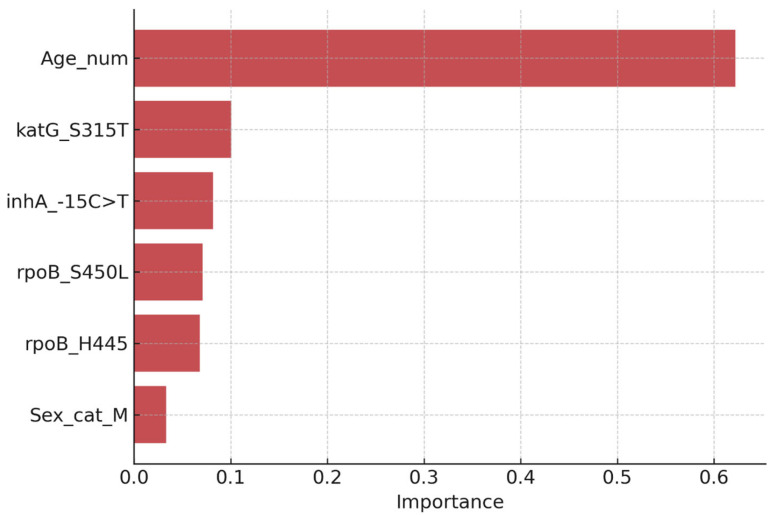
Random forest feature importance for predictors of drug resistance.

Random Forest analysis yielded comparable discrimination and ranked genetic mutations as the most influential predictors of resistance (Figure 2). The highest feature importance values were observed for *rpoB* S450L, *katG* S315T, and *inhA* −15C>T. In contrast, demographic variables (age and sex) contributed minimally to classification performance.

Feature importance values were derived from the mean decrease in Gini impurity across 500 trees, averaged across cross-validation folds.

Random Forest feature importance ranking for predictors of isoniazid and/or rifampicin resistance. Genetic mutations (*rpoB* S450L, *katG* S315T, and *inhA* −15C>T) were the strongest contributors to model classification, while demographic variables (age and sex) showed comparatively low importance.

#### Logistic Regression Predictors of Resistance

Adjusted odds ratios (Table 4) indicated that genetic mutations were the dominant predictors of isoniazid and/or rifampicin resistance. The presence of *katG* S315T was associated with a 2.85-fold increase in the odds of resistance (95% CI: 1.40–5.80). The *inhA* promoter mutation −15C>T conferred a 1.95-fold increase (95% CI: 1.05–3.60). The *rpoB* S450L mutation was the strongest single predictor, increasing the odds of resistance more than fourfold (aOR: 4.20; 95% CI: 2.10–8.45).

### 3.4. Predictive Risk Stratification

Model-derived probability estimates were used to stratify patients into three risk categories for isoniazid and/or rifampicin resistance:

- Low risk: <10% predicted probability
- Moderate risk: 10–30% predicted probability
- High risk: >30% predicted probability

**Figure 3:**
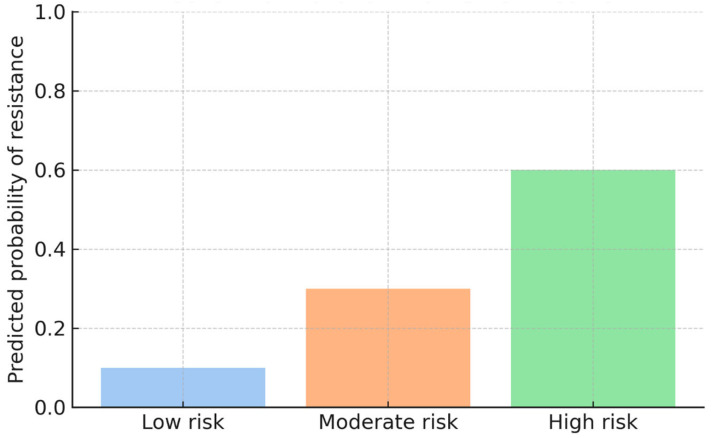
Risk stratification of patients based on model-derived predicted probabilities of isoniazid and/or rifampicin resistance. Probability thresholds were defined as low risk (<10%), moderate risk (10–30%), and high risk (>30%).

Based on these thresholds, 18% of patients were classified as high risk, 27% as moderate risk, and 55% as low risk (Figure 3). High-risk patients demonstrated a substantially greater prevalence of resistance-associated mutations, supporting the discriminatory capacity of the stratification framework.

Figure 3 illustrates the distribution of predicted probabilities across the three risk categories and shows how the model distinguishes cases based on their estimated likelihood of harbouring drug-resistant *Mycobacterium tuberculosis*. This probability-based stratification provides a structured basis for prioritizing diagnostic evaluation and potential early intervention.

#### Predictive Risk Stratification and Operational Implications

Together, these findings suggest that predictive risk stratification can identify patients at elevated risk of drug resistance and may provide a structured basis for prioritizing diagnostic and programmatic interventions. When combined with targeted molecular testing and adherence support strategies, such stratification could potentially reduce diagnostic delays, improve regimen alignment, and support more efficient allocation of limited diagnostic and community health resources. To illustrate possible programmatic implications, predicted risk categories were mapped to hypothetical intervention strategies and expected outcomes under defined operational assumptions (Table 5).

### 3.5. Scenario-Based Operational Modelling

Scenario modelling was conducted to examine the potential programmatic effects of risk-guided intervention strategies under predefined operational assumptions. For the high-risk group, prioritizing reflex LPA testing was projected to reduce the median time to start an appropriate treatment from around 14 days to about 3 days. For the moderate-risk group, prioritizing faster molecular testing was estimated to cut diagnostic delays by roughly 30%. Additionally, improved adherence counselling in this group was projected to increase treatment adherence by approximately 10–12%. Under the modeled assumptions, the combined use of predictive risk stratification and reflex testing was estimated to reduce progression from isoniazid-resistant TB to MDR-TB by about 12–15%. These estimates are deterministic projections based on simplified operational assumptions and should be viewed as theoretical programmatic estimates rather than observed clinical outcomes.

#### Predictive Risk Stratification and Projected Operational Implications

The predictive modelling pipeline classified patients into low-, moderate-, and high-risk groups for isoniazid and/or rifampicin resistance and MDR-TB. Logistic regression demonstrated strong discriminatory ability, with AUC values of 0.96 for INH and/or RIF resistance and 0.99 for MDR-TB. Random Forest classifiers produced similar results and consistently identified *katG* S315T, *inhA* −15C>T, and *rpoB* S450L as the top predictors. Based on predicted probabilities, patients were divided into three risk categories: low risk (<10%), moderate risk (10–30%), and high risk (>30%). In the study group, 18% of patients were categorized as high risk, 27% as moderate risk, and 55% as low risk. High-risk patients showed a significantly higher occurrence of resistance mutations, especially *katG* S315T and *rpoB* mutations, confirming the effectiveness of the stratification thresholds.

To evaluate operational implications, scenario-based modelling assessed how risk-guided actions could influence diagnostic and program outcomes under certain assumptadions. For the high-risk group, prioritizing reflex LPA testing and immediate treatment review was projected to reduce the median time to start appropriate treatment from around 14 days to 3 days. Targeted household screening for high-risk cases was also estimated to improve early detection of secondary TB infections by approximately 20%, potentially decreasing onward transmission.

For the moderate-risk group, faster molecular testing within 72 h was estimated to cut diagnostic delays by about 30% compared to standard procedures. Enhanced counselling and follow-up support in this group were expected to increase treatment adherence by 10–12%. For the low-risk group: patients with a resistance prevalence below 5% were recommended to maintain the standard diagnostic process while optimizing the use of molecular testing resources.

Under these modelled assumptions, combining predictive risk classification with reflex LPA testing for high-risk patients was estimated to reduce the progression from isoniazid-resistant TB to MDR-TB by approximately 12–15%. Incorporating community education interventions based on risk tiers was projected to further lower acquired resistance by improving adherence among moderate- and high-risk patients. These projections are deterministic estimates based on simplified operational assumptions and should be viewed as theoretical program scenarios rather than actual clinical outcomes.

### 3.6. Conceptual Integration of Clinical Governance and Community Engagement

Model-derived risk categories were mapped onto predefined CG structures and CEE strategies to demonstrate potential operational pathways.

**Figure 4:**
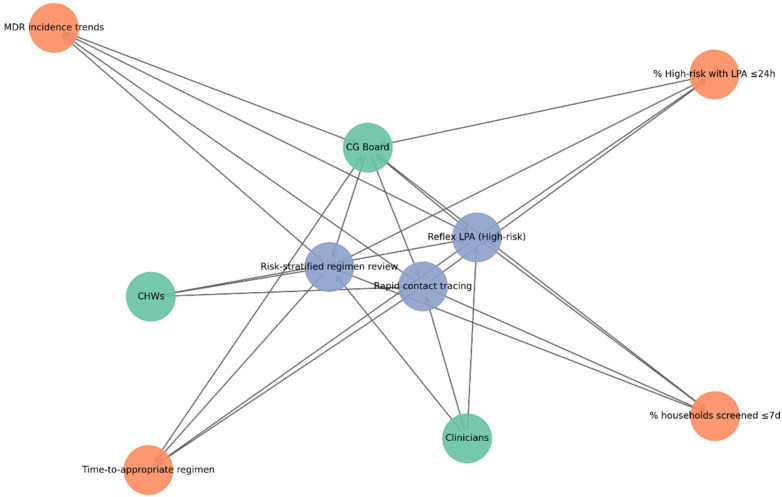
Clinical governance network illustrating relationships between operational actors, SOPs, and KPIs within the TB control framework.

Within CG systems, high-risk classification was conceptually linked to reflex LPA testing, expedited regimen review, and monitoring of KPIs, including time to appropriate regimen initiation and household screening coverage (Figure 4). The governance network (Figure 4) shows how CG structures connect operational actors, procedures, and monitoring metrics. Green nodes represent key actors (CG Board, clinicians, and community health workers), blue nodes indicate SOPs such as reflex LPA testing, risk-stratified regimen review, and rapid contact tracing, and orange nodes depict KPIs, including the proportion of high-risk patients receiving LPA within 24 h, time to appropriate regimen initiation, household screening coverage within seven days, and trends in MDR-TB incidence. Directed connections show accountability pathways where the CG Board establishes SOPs, clinicians and CHWs carry them out, and operational outputs produce KPI data that are reported back to governance structures. This setup illustrates a closed audit–feedback loop in which governance oversight, clinical implementation, and performance monitoring work as interconnected components of TB program management. It is important to note that the CG–CEE integration presented here is a conceptual operational framework derived from model results, rather than a prospectively evaluated implementation strategy.

#### 3.6.1. Integration with Clinical Governance

The CG framework embeds ML outputs into routine decision-making processes. Key SOPs include reflex LPA testing for high-risk cases, risk-stratified regimen review, and rapid contact tracing. Programmatic monitoring is supported by KPIs, including time to appropriate regimen initiation and household screening coverage.

The governance network (Figure 4) illustrates how operational actors, procedures, and monitoring metrics interact within the TB control system. Green nodes represent key actors (CG Board, clinicians, and community health workers), blue nodes denote SOPs, and orange nodes represent KPIs. Directed edges illustrate accountability pathways in which the CG Board establishes SOPs, clinicians and CHWs implement them, and resulting operational outputs generate KPI data that are reported back to governance structures.

This configuration illustrates a closed audit–feedback loop linking governance oversight, clinical implementation, and programme monitoring. The CG–CEE integration presented here represents a conceptual operational framework derived from model outputs rather than a prospectively evaluated implementation strategy, illustrating how mutation-informed predictive models could be incorporated into existing TB governance systems.

#### 3.6.2. Integration with Community-Engaged Education

**Figure 5:**
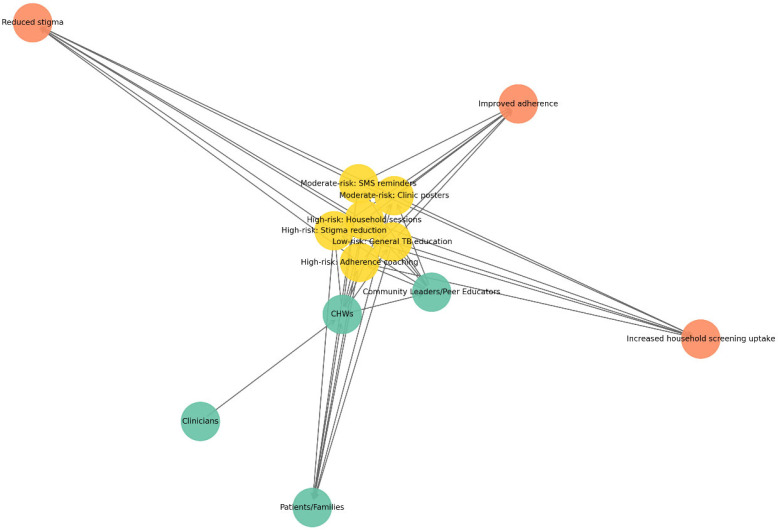
Network representation of risk-stratified CEE interventions linking community actors and anticipated programmatic outcomes.

CEE interventions were aligned with the risk bands identified by the predictive models. High-risk patients and their households were conceptually associated with intensified education and adherence support, including household counselling, stigma-reduction activities, and adherence coaching delivered by CHWs. Moderate-risk cases received reinforced adherence support via SMS or WhatsApp reminders and clinic-based education materials, while low-risk groups received routine TB education. The community network model (Figure 5) illustrates how risk-stratified interventions interact with community and health-system actors. Green nodes represent actors (CHWs, clinicians, patients and families, and community leaders), yellow nodes represent interventions stratified by risk level, and orange nodes represent anticipated outcomes, including improved treatment adherence, reduced stigma, and increased household screening uptake. Directed connections illustrate how CHWs function as central agents linking clinical services, households, and community leadership structures. This network highlights the role of community-based engagement in reinforcing adherence, reducing stigma, and supporting household screening, complementing governance-based interventions within the TB control framework.

#### 3.6.3. Systems Integration of Clinical Governance and Community Engagement

**Figure 6:**
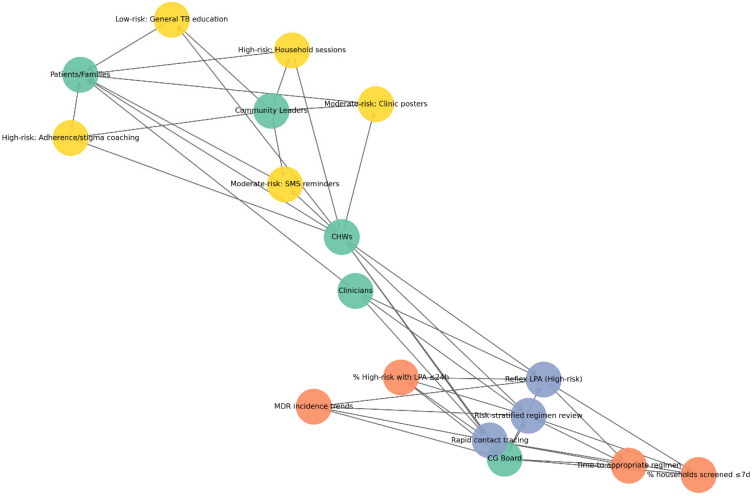
The systems map illustrates the integration of CG processes and CEE interventions within the TB control framework.

The systems map (Figure 6; Table 6) illustrates the interconnected roles of CG and CEE in strengthening TB control. Nodes are grouped into four functional categories: actors, governance processes, monitoring indicators, and community interventions.

Actor nodes include clinicians, CHWs, the CG Board or morbidity-and-mortality (M&M) committee, patients and families, and community leaders or peer educators. Governance process nodes represent SOPs, including reflex LPA testing for high-risk cases, risk-stratified regimen review, and rapid contact tracing. Monitoring nodes correspond to KPIs, including the proportion of high-risk patients receiving LPAs within 24 h, time to appropriate regimen initiation, household screening coverage within seven days, and trends in MDR-TB incidence. Community intervention nodes represent CEE strategies stratified by risk level, including household education sessions, stigma reduction and adherence coaching for high-risk households, SMS or WhatsApp reminders, and clinic posters for moderate-risk groups, and general TB education for low-risk populations. Directed edges represent accountability, operational, and feedback relationships across the system. Accountability links connect the CG Board with SOP implementation and KPI monitoring, while operational links demonstrate collaboration between clinicians and CHWs through two-way risk communication. Community links depict CHWs delivering education and support to households and working with community leaders. Feedback pathways show how barriers to adherence or service delivery identified at the community level are reported back to governance structures. Overall, the network emphasizes CHWs as key connectors, linking governance oversight with community action and maintaining accountability loops among policy, clinical practice, and patient education.

## 4. Discussion

These findings should be interpreted as proof-of-concept for mutation-informed risk stratification rather than as a finalized deployable predictive tool. Although the internally validated performance of the predictive models was strong, the framework should be interpreted as proof-of-concept derived from a single regional dataset. External validation across independent populations and prospective temporal validation will be necessary to confirm the model’s generalizability and operational feasibility before routine implementation. The proposed integration of predictive risk stratification into CG and CEE structures should likewise be interpreted as a hypothesis-generating operational model. Although the framework was informed by existing TB program workflows, it has not yet been prospectively evaluated through implementation or feasibility studies. Future research should therefore include stakeholder consultations, pilot implementation, and mixed-methods evaluation to assess feasibility, acceptability, and real-world effectiveness within routine TB control programmes. The study focused on a limited number of high-frequency resistance-associated mutations. While this may not capture the full genomic diversity of *Mycobacterium tuberculosis*, it reflects routine diagnostic practice and priorities clinically actionable mutations. Future studies incorporating whole-genome sequencing data could provide a more comprehensive understanding of resistance mechanisms. Although previous studies have reported higher predictive performance and more comprehensive mutation coverage, many of these analyses were conducted in well-resourced settings using larger datasets and advanced genomic platforms such as whole-genome sequencing. In contrast, the present study was designed to reflect real-world programmatic conditions in a high-burden, resource-limited setting. The use of routinely available molecular diagnostics and a parsimonious modelling approach enhances the practical applicability and scalability of the findings. Therefore, rather than directly competing with existing models, this study provides complementary, context-specific insights relevant for operational use in TB control programs.

### 4.1. Phenotype and Mutation Analysis

The high prevalence of resistance phenotypes (58.9%) observed in this cohort underscores the continuing burden of DR-TB in the study setting. MDR-TB accounted for 21.7% of cases, which is comparable to observations from other high-burden settings and reinforces the need for strengthened diagnostic and treatment strategies [27,28]. Mutation analysis demonstrated patterns consistent with globally established resistance mechanisms. The *katG* S315T mutation was the most frequently detected isoniazid resistance-associated variant, confirming its well-documented role as a major determinant of high-level INH resistance [8,9]. Promoter mutations in *inhA*, particularly −15C>T, were also detected and occasionally co-occurred with *katG* mutations. This observation reflects the recognized molecular complexity of INH resistance and its potential implications for treatment optimization and resistance interpretation [10,11]. Among the rifampicin resistance-associated mutations evaluated in this study (*S450L*, *H445*, and *D435* variants), *rpoB* S450L was the most frequently detected variant. This observation is consistent with global evidence indicating that S450L is one of the most common high-confidence rifampicin resistance mutations [8,12]. Importantly, this comparison is limited to the mutation spectrum assessed in the present dataset and does not imply evaluation of all variants described in the WHO mutation catalogue. The strong concordance between mutation profiles and phenotypic resistance supports the continued use of rapid molecular diagnostic platforms, such as LPAs and other WHO-recommended tests, for early resistance detection [13,14]. The observed enrichment of combined *katG* and *inhA* mutations among MDR isolates suggests that reliance on a single molecular marker may underestimate resistance risk in certain contexts. From a programmatic perspective, these findings highlight the importance of integrating mutation-level data into timely clinical decision-making and of strengthening CG mechanisms to ensure rapid treatment modification and support adherence [16,17]. Taken together, the convergence of phenotypic and genotypic findings reinforces the reliability of canonical molecular resistance markers and supports their continued use within structured diagnostic algorithms for TB control programs [15,29].

### 4.2. Diagnostic Machine Learning Analysis

The application of diagnostic machine learning approaches provided complementary insights into predictors of drug resistance. Logistic regression identified resistance-associated mutations, particularly *rpoB* S450L and *katG* S315T, as the strongest independent predictors of resistance. The Random Forest classifier yielded comparable discrimination and similarly ranked genetic variables as dominant predictors, reinforcing the biological centrality of these mutations.

The strong internally validated discrimination observed in this study (AUCs of 0.96 for INH and/or RIF resistance and 0.99 for MDR-TB) likely reflects the mechanistic relationship between canonical resistance mutations and phenotypic drug resistance. In this biologically deterministic context, complex nonlinear modelling approaches were not expected to substantially outperform logistic regression. Comparable observations have been reported in previous studies. For instance, Babirye et al. [19] demonstrated the feasibility of ML-based resistance prediction using *Mycobacterium tuberculosis* genomic data. Conversely, other investigations have reported minimal performance differences between machine learning algorithms and traditional statistical models when predictor sets are limited and biologically well-defined [23]. These findings suggest that, in resource-constrained settings, mutation-based molecular diagnostics may provide greater programmatic value than demographic risk profiling alone. However, although internal cross-validation supported model stability, external validation in independent populations remains essential before these predictive models can be deployed in routine clinical care [30]. Furthermore, interpretability remains critical for adoption in clinical settings, underscoring the continued practical value of logistic regression in providing transparent effect estimates alongside ML-based robustness assessments.

### 4.3. Predictive Risk Stratification and Resource Targeting

The predictive modelling framework presented in this study provides a forward-looking approach to TB management, linking individual-level risk probabilities with operational decision-making. By stratifying patients into low-, moderate-, and high-risk categories, the model advances beyond descriptive resistance profiling and provides actionable guidance for clinicians and TB program managers. For high-risk patients, the model highlights the importance of reflex molecular testing and prompt review of treatment regimens [20,21,30]. Scenario-based modelling suggested that this approach could substantially reduce time to appropriate therapy, a critical factor for preventing amplification of resistance and limiting household and community transmission. Targeted contact tracing and household education triggered by high-risk classification may further contribute to early interruption of transmission chains. Moderate-risk patients may benefit from expedited molecular testing and enhanced adherence support. Delays in confirming resistance or inconsistent treatment adherence often contribute to poor outcomes within this group. By directing enhanced counselling and monitoring toward moderate-risk patients, TB programs may reduce diagnostic delays, improve treatment adherence, and lower the probability of acquired resistance. Low-risk patients, who comprised the majority of cases in this cohort, reinforce the framework’s efficiency. Their classification supports prioritization of scarce molecular diagnostic resources and intensive adherence interventions for higher-risk groups, while maintaining access to standard TB diagnostic pathways and routine health education [22,31,32]. Importantly, the predictive framework also strengthens the connection between CG systems and community engagement mechanisms. Risk thresholds can be incorporated into CG structures as quality indicators, supporting accountability for timely reflex testing and household screening. At the same time, CEE strategies can be stratified by patient risk categories, ensuring that high-risk households receive intensive support while moderate- and low-risk households benefit from scalable community-level interventions. Overall, these findings indicate that ML-supported risk stratification could help reduce diagnostic delays, improve resource allocation, and strengthen both clinical and community-based TB control efforts. However, further validation in larger, more diverse populations, along with the inclusion of additional clinical predictors such as HIV status, prior treatment history, and relevant comorbidities, will be necessary to confirm the scalability and applicability of this approach. The integrated systems framework shown in Figure 6 demonstrates how mutation-informed risk stratification could be implemented through coordinated CG and CEE mechanisms within routine TB control programs.

### 4.4. Strengths and Limitations

This study integrates molecular, clinical, and programmatic data within a real-world, high-burden rural setting, thereby enhancing the practical relevance and translational value of the findings. The use of routinely collected diagnostic and surveillance data strengthens external applicability to similar TB program environments. Furthermore, the modeling approach was intentionally parsimonious, focusing on biologically well-established predictors, which improves interpretability while reducing the risk of overfitting. Internal validation using stratified 10-fold cross-validation further enhanced model robustness by assessing stability and minimising optimism bias in performance estimates.

Notwithstanding these strengths, several limitations should be considered. First, the study was conducted within a single provincial cohort in the Eastern Cape, South Africa, which may limit the generalisability of the findings to settings with different epidemiological profiles, mutation distributions, or health system capacities. Although the sample size (n = 207, including 45 MDR-TB cases) was adequate for parsimonious modeling, it remains modest for predictive model development. Consequently, performance estimates derived from internal validation may be optimistic and require confirmation through external validation in independent populations. Secondly, the mutation spectrum was restricted to high-confidence variants detectable through routine diagnostic platforms available in the study setting. While this enhances clinical applicability, it does not capture the full genomic diversity of *Mycobacterium tuberculosis*, particularly rare or emerging mutations. This was a deliberate methodological choice to prioritise model stability and operational relevance; however, future studies incorporating whole-genome sequencing could provide a more comprehensive understanding of resistance mechanisms. Thirdly, although internal cross-validation was applied to mitigate overfitting, the relatively small dataset may still be sensitive to sampling variability, particularly when using flexible machine learning algorithms such as Random Forests. As such, the model should be regarded as internally validated, and further evaluation using larger, multi-site datasets across different time periods is necessary to assess calibration, transportability, and real-world performance. Fourthly, the scenario-based projections examining reductions in diagnostic delay and MDR-TB progression were based on deterministic assumptions and did not incorporate uncertainty analyses or dynamic transmission modelling. These projections should therefore be interpreted as illustrative rather than predictive of real-world intervention effects. Finally, treatment outcomes were aggregated into a binary classification: successful versus unsuccessful. While this approach improved statistical power and model stability, it may obscure important differences between outcome categories such as death, treatment failure, and loss to follow-up, which are driven by distinct clinical and social factors. Future research with larger datasets should explore disaggregated or multinomial modelling approaches to better capture these pathways.

## 5. Conclusions

This study shows that mutation-informed predictive modeling can effectively distinguish isoniazid and/or rifampicin resistance and MDR-TB through strong internal validation. Using a streamlined set of predictors based on well-known resistance-conferring mutations, the models demonstrated consistent performance within the study dataset. Logistic regression provided clear effect estimates suitable for clinical interpretation, while ML approaches mainly served as robustness checks rather than offering significant predictive improvements. Importantly, these findings should be viewed as a proof-of-concept based on a single regional cohort. Although internal cross-validation indicated model stability, external and temporal validation in independent populations will be necessary to confirm model calibration, transferability, and practical use before adopting it routinely. Beyond predictive modelling, this study proposes a governance-linked framework that combines molecular diagnostics with risk-based clinical pathways and community-centred education strategies. This systems-focused approach shows how predictive outputs could be integrated into existing TB program structures to support reflex diagnostic testing, reduce delays in treatment initiation, and improve adherence support in resource-limited settings. In high-burden rural areas like the Eastern Cape, mutation-informed risk stratification might offer a practical way to improve TB program responsiveness and optimize limited diagnostic resources. Future research should focus on prospective validation, multi-centre replication, and implementation studies to assess real-world effectiveness and uncertainties. Adding more clinical and behavioral predictors could further enhance model performance and operational usefulness.

## Figures and Tables

**Table 1:** Distribution of drug-resistance phenotypes among *Mycobacterium tuberculosis* isolates (n = 207).

Phenotype	n (%)
Susceptible	85 (41.1)
INH and/or RIF resistance	122 (58.9)
MDR-TB	45 (21.7)
RIF monoresistant	28 (13.5)
INH monoresistant	36 (17.4)

Abbreviations: INH, isoniazid; RIF, rifampicin; MDR-TB, multidrug-resistant tuberculosis (resistance to both isoniazid and rifampicin).

**Table 2:** Prevalence of resistance-associated mutations among *Mycobacterium tuberculosis* isolates (n = 207).

Mutation	n (%)
*katG* S315T	60 (29.0)
*inhA* −15C>T	42 (20.3)
*rpoB* S450L	55 (26.6)
*rpoB* H445 variants	18 (8.7)
*rpoB* D435 variants	12 (5.8)

**Table 3:** Common mutation combinations and associated resistance phenotypes.

Mutation Combination	Associated Phenotype	n (%)
*katG* S315T + *inhA* −15C>T	Isoniazid-resistant TB/MDR-TB	28 (13.5)
*rpoB* S450L	Rifampicin-resistant TB/MDR-TB	40 (19.3)
*katG* S315T only	Isoniazid monoresistant	22 (10.6)
*inhA* −15C>T only	Isoniazid monoresistant	14 (6.8)
No mutation detected	Susceptible	85 (41.1)

**Table 4:** Adjusted odds ratios for predictors of isoniazid and/or rifampicin resistance.

Predictor	Adjusted OR	95% CI
*katG* S315T	2.85	1.40–5.80
*inhA* −15C>T	1.95	1.05–3.60
*rpoB* S450L	4.20	2.10–8.45
Age (per 10-year increase)	1.10	0.90–1.35
Sex (male vs. female)	1.20	0.75–1.95

**Table 5:** Predictive risk bands, proposed interventions, and projected operational outcomes.

Risk Band	% Patients (n = 207)	Proposed Interventions	Projected Operational Outcomes *
High-risk (>30%)	18%	Reflex LPA testing; immediate regimen review; prioritized household screening.	Reduced median time to appropriate regimen (14 → ~3 days); increased early detection of secondary cases (~20%); potential reduction in progression from isoniazid-resistant TB to MDR-TB (~12–15%).
Moderate-risk (10–30%)	27%	Expedited molecular testing (≤72 h); adherence counselling; scheduled follow-up.	Reduced diagnostic delay (~30%); improved treatment adherence (~10–12%); strengthened linkage to care.
Low-risk (<10%)	55%	Standard diagnostic pathway; routine TB education.	Efficient allocation of molecular diagnostics; stable outcomes with low baseline resistance prevalence.

* Projected outcomes represent deterministic scenario-based estimates under predefined implementation assumptions and should not be interpreted as observed intervention effects.

**Table 6:** Nodes included in the CG–CEE systems network illustrating governance structures, monitoring indicators, and community-level interventions for tuberculosis control.

Category	Nodes
Actor	Clinicians
Actor	CHWs
Actor	CG Board
Actor	Patients/Families
Actor	Community Leaders
CG SOP	Reflex LPA (high risk)
CG SOP	Risk-stratified regimen review
CG SOP	Rapid contact tracing
KPI	% high-risk with LPA ≤24 h
KPI	Time-to-appropriate regimen
KPI	% households screened ≤7 d
KPI	MDR incidence trends
CEE intervention	High risk: household sessions
CEE intervention	High risk: adherence/stigma coaching
CEE intervention	Moderate risk: SMS reminders
CEE intervention	Moderate risk: clinic posters
CEE intervention	Low risk: general TB education

## Data Availability

The datasets generated and analyzed during the current study are not publicly available due to data protection regulations of the Eastern Cape Department of Health and the National Health Laboratory Service. De-identified aggregated data may be made available from the corresponding author upon reasonable request and with permission of the respective data custodians.

## Abbreviations

The following abbreviations are used in this manuscript:

aORs	adjusted odd ratios
CG	Clinical Governance
CEE	Community-engaged Education
CHWs	Community Health Workers
DHIS	District Health Information System
DR-TB	Drug-resistant tuberculosis
EPV	Events-per-variable
INH	Isoniazid
KPIs	Key performance indicators
LPA(s)	Line Probe Assay(s)
ML	Machine learning
M&M	Morbidity and Mortality
MDR-TB	Multidrug-resistant tuberculosis
ROC	Receiver operating characteristics
RIF	Rifampicin
RRDR	Rifampicin resistance-determining region
SOPs	Standard Operating Procedures
TRIPOD	Transparent Reporting of a multivariable prediction model for Individual Prognosis or Diagnosis
TB	Tuberculosis
VIFs	Variance inflation factors
WHO	World Health Organization
